# Reply to the Letter to the Editor regarding “Therapeutic plasma exchange in acute liver failure: Unravelling its efficacy and impact”

**DOI:** 10.1016/j.htct.2026.106515

**Published:** 2026-08-28

**Authors:** Sonal Sonu, Rajesh Kumar

**Affiliations:** a All India Institute of Medical Sciences, Bathinda, Punjab, India; b Dayanand Medical College and Hospital, Ludhiana , Punjab

To the Editor,

We appreciate the opportunity to address the comments regarding our recently published article, “Therapeutic plasma exchange in acute liver failure: Unravelling its efficacy and impact.” [1]

We value the thoughtful appraisal of our work and agree that several important methodological considerations inherent to retrospective observational studies merit acknowledgment. [2] Our study was primarily intended to evaluate the feasibility, biochemical response, and practical applicability of therapeutic plasma exchange (TPE) in patients with acute liver failure (ALF) managed in a resource-constrained setting without access to liver transplantation services.

We acknowledge that the absence of a concurrent control group limits definitive causal inference regarding treatment efficacy. However, the objective of our study was not to establish superiority comparable to randomized controlled trials, but rather to provide real-world clinical data from a setting where therapeutic options remain limited. In critically ill ALF patients, retrospective analyses continue to provide clinically relevant evidence, particularly from centers where transplantation facilities are unavailable.

We also recognize the limitations associated with the relatively small sample size and multiple statistical comparisons. The analyses were exploratory in nature and intended to generate preliminary observations that may support future prospective investigations. We agree that larger multicentric studies with appropriate statistical adjustment would strengthen the available evidence.

The observation that biochemical improvement was more pronounced among discharged patients than non-survivors may indeed reflect differences in baseline disease severity and survivor bias, as appropriately highlighted. Nevertheless, we believe these findings remain clinically informative, particularly given the limited regional data evaluating TPE outcomes in ALF. Data regarding TPE outcomes in ALF from Indian and other resource-constrained settings remain limited, and we believe such observational evidence contributes meaningfully to the existing literature. Importantly, our conclusions were intended to emphasize association and clinical applicability rather than establish definitive therapeutic efficacy.

Regarding long-term clinical endpoints, including transplantation-free survival and encephalopathy resolution, comprehensive follow-up data were limited by the retrospective design and incomplete follow-up for some patients, particularly those discharged against medical advice. Our reference to TPE as a potential bridge to transplantation was based on existing literature and current therapeutic rationale rather than direct transplantation outcomes from our institution.

With respect to safety reporting, adverse events were documented on a per-patient basis. We agree that standardized procedure-wise reporting and formal complication grading systems would improve comparability across studies and should be incorporated in future analyses.

We value the constructive suggestions provided, including the possibility of propensity-matched analyses and subgroup stratification, which may serve as valuable directions for future prospective research.

Despite the acknowledged limitations inherent to retrospective observational studies, we believe our findings provide clinically relevant preliminary evidence supporting the feasibility and acceptable safety profile of TPE in ALF patients managed in resource-constrained settings where immediate access to transplantation may not be feasible.

We once again thank the Editor for facilitating this valuable academic discussion. Sincerely,

Sonal Sonu et al.

## Funding

No external funding was received for this correspondence.

## Author contributions

All authors contributed to the drafting, review, and approval of the final manuscript.

## Conflicts of interest

The authors declare no conflict of interest.
